# WHO Air Quality Guidelines Need to be Adopted

**DOI:** 10.3389/ijph.2021.1604483

**Published:** 2021-10-15

**Authors:** Heresh Amini

**Affiliations:** Department of Public Health, University of Copenhagen, Copenhagen, Denmark

**Keywords:** air pollution, conformity, particulate matter, nitrogen dioxide, ozone

It is very well known that air pollution causes death, and a wide spectrum of health conditions, with considerable burden for the world’s population [1]. There is evidence also for the association of air pollution with SARS-CoV-2 transmission, COVID-19 infection severity, and its mortality [2–4].

The World Health Organization (WHO) has launched air quality guidelines (AQG) 2021 about 15 years after 2005 AQGs for short- and long-term exposure to a range of air pollutants, such as particulate matter (PM_2.5_ and PM_10_), ozone (O_3_), nitrogen dioxide (NO_2_), sulfur dioxide (SO_2_) and carbon monoxide (CO) [5]. In brief, most 2021 AQGs are lower compared to 2005 using updated WHO methodology, and given the fact that new evidence shows health effects occur even at lower exposure levels [6–8]. The updated WHO AQGs are based on thorough systematic reviews and meta-analyses of evidence up to mid-2020 [5].

Of notable updates, the 2021 AQG compared to 2005 for annual mean exposure to PM_2.5_ reduced from 10 to 5 µg/m^3^, PM_10_ reduced from 20 to 15, and NO_2_ reduced from 40 to 10 µg/m^3^. Furthermore, there is new 2021 AQG for peak season O_3_ (60 µg/m^3^) and 24-hour exposure to CO (4 mg/m^3^). The AQG for 24-hour exposure to SO_2_ increased from 20 µg/m^3^ in 2005 to 40 µg/m^3^ in 2021, which is due to updated evidence and methodology for AQGs. Amongst other updates, the 2021 AQGs also provide good practice statements for certain types of PM, such as black carbon/elemental carbon, ultrafine particles, and dust- and sandstorms. It is notable that WHO AQGs further provide Interim Targets (ITs) for most pollutants for stepwise progress towards achieving the AQGs. As WHO emphasized, it is very important to note that the 2005 WHO AQGs remain valid for pollutants and those averaging times not covered in 2021 update [5].

As shown in Figure 1, it is evident that there is inequality in exposure to air pollution across the world with low- and middle-income countries (LMICs) experiencing higher exposure levels for most pollutants. Currently, large world populated areas do not meet the WHO AQG 2021 for annual mean exposure to PM_2.5_, annual mean NO_2_, and seasonal maximum O_3_, and many countries are even in a position that need to consider IT1 for PM_2.5_ (35 µg/m^3^) as the first step to achieve, which is indeed challenging. NO_2_ is considerably higher within urban areas, and ground level O_3_ has high values across the Middle East and India (Figure 1).

**FIGURE 1:**
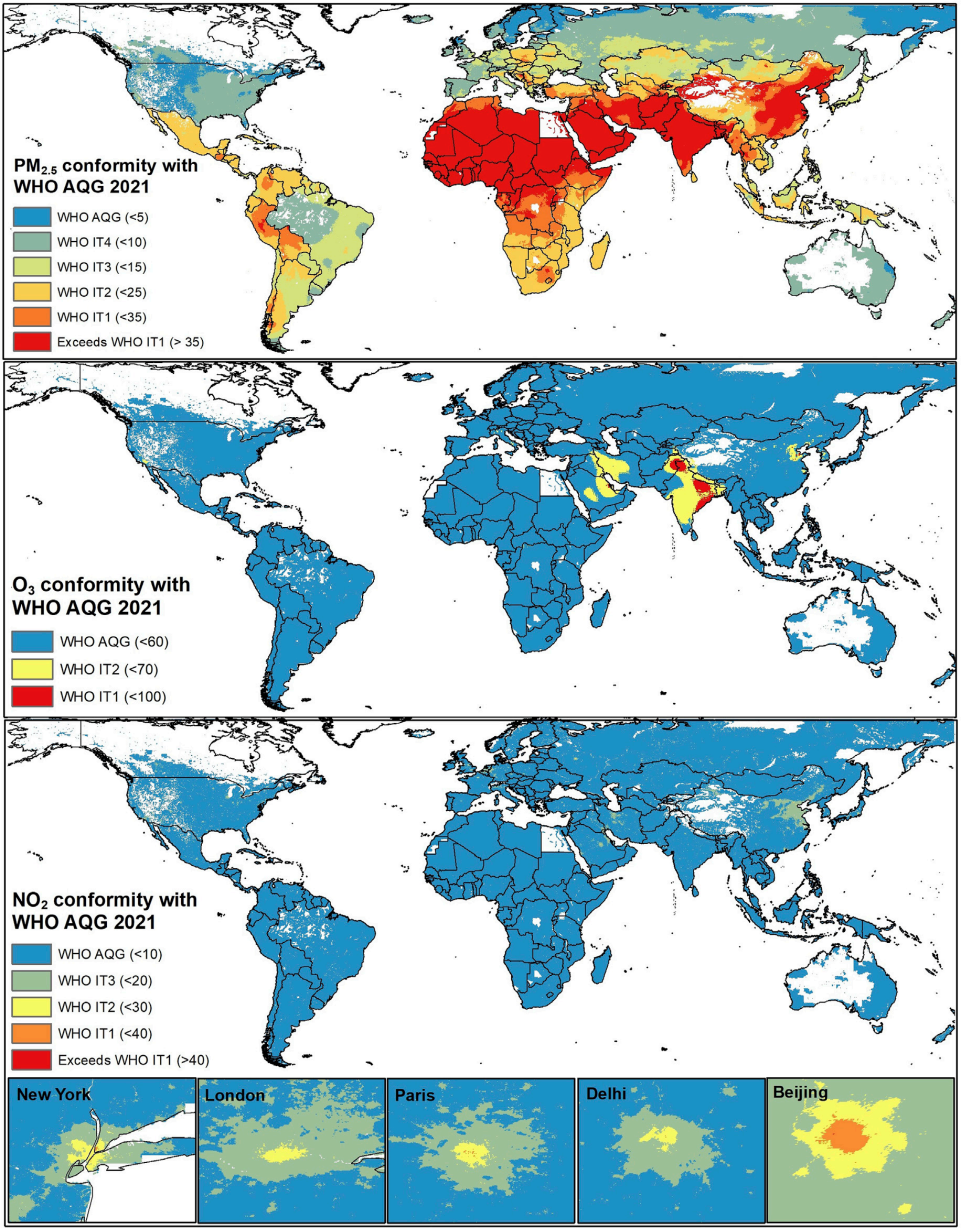
The conformity of 2019 annual mean PM_2.5_, 2019 seasonal maximum O_3_, and 2011 annual mean NO_2_ with the WHO Air Quality Guidelines (AQG) 2021 and interim targets (IT) across the world. Human populations do not populate the white areas within the countries. Source: PM_2.5_ adapted from Shaddick et al. (2018) [9] and Global Burden of Disease (GBD) 2019 Risk Factor Collaborators [1, 9], O_3_ adapted from Chang et al. (2019) and GBD 2019 Risk Factor Collaborators [1, 10], and NO_2_ adapted from Larkin et al. (2017) [11].

The WHO AQGs 2021 have important implications for WHO member states and public health. With the launch of WHO AQGs 2021, WHO has provided the member states with a tool that need to be adopted to protect public health from air pollution as a so-called “silent killer.” As stated in a joint statement by Hoffmann et al. [12], which is endorsed by more than hundred medical, public health, scientific and patient representative societies, such as European Respiratory Society (ERS) and the International Society of Environmental Epidemiology (ISEE), immediate action is needed to use these guidelines for emission reduction policy making and adopt these science-based guidelines and interim targets as national air quality standards. Clearly, healthy lungs and healthy hearts need clean air [3, 13].

It is not only the duty of governments to achieve these goals, but also it is a societal responsibility for all individuals to move in the right direction and reduce the unacceptable burden of air pollution (millions of lost lives, years of life disabled and lost, and related costs). The extent of the reduction is directly translated into improvement in public health. This require actions that are in line with climate action as one of the 17 Sustainable Development Goals established by the United Nations in 2015 [14].

## References

[1] Murray CJL Aravkin AY Zheng P Abbafati C Abbas KM Abbasi-Kangevari M. Global burden of 87 Risk Factors in 204 Countries and Territories, 1990-2019: a Systematic Analysis for the Global Burden of Disease Study 2019. Lancet (2020) 396(10258):1223–49. 10.1016/S0140-6736(20)30752-2 33069327 PMC7566194

[2] Xu R Rahmandad H Gupta M DiGennaro C Ghaffarzadegan N Amini H.Weather, Air Pollution, and SARS-CoV-2 Transmission: a Global Analysis. Lancet Planet Health (2021) 5(10):e671–e80. 10.1016/S2542-5196(21)00202-3 34627471 PMC8497024

[3] Brauer M Casadei B Harrington RA Kovacs R Sliwa K Davaakhuu N. Taking a Stand against Air Pollution - the Impact on Cardiovascular Disease. Eur Heart J (2021) 42(15):1460–3. 10.1093/eurheartj/ehaa1025 33507239 PMC7953955

[4] Wu X Nethery RC Sabath MB Braun D Dominici F. Air Pollution and COVID-19 Mortality in the United States: Strengths and Limitations of an Ecological Regression Analysis. Sci Adv (2020) 6(45):eabd4049. 10.1126/sciadv.abd4049 33148655 PMC7673673

[5] World Health Organization. WHO Global Air Quality Guidelines: Particulate Matter (PM2.5 and PM10), Ozone, Nitrogen Dioxide, Sulfur Dioxide and Carbon Monoxide. Geneva: World Health Organization. (2021). 34662007

[6] Dominici F Schwartz J Di Q Braun D Choirat C Zanobetti A. Assessing Adverse Health Effects of Long-Term Exposure to Low Levels of Ambient Air Pollution: Phase 1. Research Report 200. Boston, MA: Health Effects Institute. (2019). PMC7300216 31909579

[7] Brunekreef B Strak M Chen J Andersen ZJ Atkinson R. Mortality and Morbidity Effects of Long-Term Exposure to Low-Level PM2.5, BC, NO2, and O3: An Analysis of European Cohorts in the ELAPSE Project. Research Report 208. Boston, MA: Health Effects Institute. (2021). PMC9476567 36106702

[8] Brauer M Brook JR Christidis T Chu Y Crouse DL Erickson A. Mortality–Air Pollution Associations in Low-Exposure Environments (MAPLE): Phase 1. Research Report 203. Boston, MA: Health Effects Institute. (2019). PMC7334864 31909580

[9] Shaddick G Thomas ML Amini H Broday D Cohen A Frostad J. Data Integration for the Assessment of Population Exposure to Ambient Air Pollution for Global burden of Disease Assessment. Environ Sci Technol (2018) 52(16):9069–78. 10.1021/acs.est.8b02864 29957991

[10] Chang K-L Cooper OR West JJ Serre ML Schultz MG Lin M. A New Method (M3Fusion V1) for Combining Observations and Multiple Model Output for an Improved Estimate of the Global Surface Ozone Distribution. Geosci Model Dev (2019) 12(3):955–78. 10.5194/gmd-12-955-2019

[11] Larkin A Geddes JA Martin RV Xiao Q Liu Y Marshall JD. Global Land Use Regression Model for Nitrogen Dioxide Air Pollution. Environ Sci Technol (2017) 51(12):6957–64. 10.1021/acs.est.7b01148 28520422 PMC5565206

[12] Hoffmann B Boogaard H de Nazelle A Andersen ZJ Abramson M Brauer M. WHO Air Quality Guidelines 2021–Aiming for Healthier Air for All: A Joint Statement by Medical, Public Health, Scientific Societies and Patient Representative Organisations. Int J Public Health (2021) 66(88). 10.3389/ijph.2021.1604465 PMC8494774 34630006

[13] Andersen ZJ Gehring U De Matteis S Melen E Vicedo-Cabrera AM Katsouyanni K. Clean Air for Healthy Lungs - an Urgent Call to Action: European Respiratory Society Position on the Launch of the WHO 2021 Air Quality Guidelines. Eur Respir J (2021) 58:2102447. 10.1183/13993003.02447-2021 34561297

[14] Lim SS Allen K Bhutta ZA Dandona L Forouzanfar MH Fullman N. Measuring the Health-Related Sustainable Development Goals in 188 Countries: a Baseline Analysis from the Global Burden of Disease Study 2015. Lancet (2016) 388(10053):1813–50. 10.1016/S0140-6736(16)31467-2 27665228 PMC5055583

